# Analysis of eIF2B bodies and their relationships with stress granules and P-bodies

**DOI:** 10.1038/s41598-018-30805-y

**Published:** 2018-08-16

**Authors:** Stephanie L. Moon, Roy Parker

**Affiliations:** 1 Department of Chemistry and Biochemistry, University of Colorado, Boulder, Colorado USA; 2 Howard Hughes Medical Institute, University of Colorado, Boulder, Colorado USA

## Abstract

Eukaryotic cells respond to stress and changes in the environment in part by repressing translation and forming cytoplasmic assemblies called stress granules and P-bodies, which harbor non-translating mRNAs and proteins. A third, but poorly understood, assembly called the eIF2B body can form and contains the eIF2B complex, an essential guanine exchange factor for the translation initiation factor eIF2. Hypomorphic *EIF2B* alleles can lead to Vanishing White Matter Disease (VWMD), a leukodystrophy that causes progressive white matter loss. An unexplored question is how eIF2B body formation is controlled and whether VWMD alleles in *EIF2B* alter the formation of eIF2B bodies, stress granules, or P-bodies. To examine these issues, we assessed eIF2B body, stress granule, and P-body induction in wild-type yeast cells and cells carrying VWMD alleles in the *EIF2B2* (*GCD7*) and *EIF2B5* (*GCD6*) subunits of eIF2B. We demonstrate eIF2B bodies are rapidly and reversibly formed independently of stress granules during acute glucose deprivation. VWMD mutations had diverse effects on stress-induced assemblies with some alleles altering eIF2B bodies, and others leading to increased P-body formation. Moreover, some VWMD-causing mutations in *GCD7* caused hyper-sensitivity to chronic GCN2 activation, consistent with VWMD mutations causing hyper-sensitivity to eIF2α phosphorylation and thereby impacting VWMD pathogenesis.

## Introduction

Translation factors play important roles in shaping the organization of the cytosol. During stress, translation suppression induces the formation and/or expansion of macromolecular complexes enriched in non-translating mRNAs, translation factors and RNA binding proteins including processing bodies (P-bodies) and stress granules (SGs)^1–4^. Because translation is a key node for regulating gene expression, increasing the local concentration of translation factors in distinct assemblies during stress could impact gene regulation by inhibiting or enhancing their function. Therefore, understanding the full set and dynamics of stress-induced complexes will be important for understanding how cells adapt to and survive stress conditions.

Another class of cytoplasmic assemblies in yeast that contain translation factors, referred to as eIF2B bodies, are less well understood. First discovered by the Ashe lab, eIF2B bodies are round or fibril-like structures that contain subunits of the eIF2B and eIF2 complexes^5–7^. Conflicting reports suggest eIF2B bodies may be constitutively present in *Saccharomyces cerevisiae* or that they are exclusively induced during long-term starvation under conditions of low cytoplasmic pH^5–8^.

EIF2B bodies are of particular interest because they contain the essential eIF2B translation initiation factor. EIF2B facilitates ternary complex formation and translation initiation through its guanine exchange activity on the eIF2 complex. Dysregulation of eIF2B or other translation factors through mutations or post-translational modifications can contribute to developmental defects, intellectual disability^9–11^ and neurodegenerative disease^12–14^. Moreover, aberrant accumulation of SGs is implicated in the pathogenesis of several neurodegenerative diseases^15,16^.

An interesting connection between eIF2B and neurodegenerative disease is that the leukodystrophy Vanishing White Matter Disease (VWMD) is caused by mutations in the genes encoding any of the five subunits of the eIF2B complex^13^. In this disease, patients undergo the progressive loss of white matter, which in some cases can be worsened and/or triggered by febrile illness or trauma^17^. However, the specific combination of molecular defects caused by the VWMD mutations and how those contribute to neurodegeneration when eIF2B is altered are not fully understood.

We hypothesized that eIF2B bodies, like SGs and P-bodies, are dynamic, inducible assemblies that form during conditions of acute stress when the cytoplasm is acidic and translation is repressed. We assessed the formation of eIF2B bodies by monitoring the localization of all five subunits of the eIF2B complex and SUI2, the yeast homolog of eIF2α. We further assessed the relationship between eIF2B bodies and SGs to determine if these assemblies co-exist and might mediate translational repression during glucose deprivation stress by the coordinated spatial segregation of translation initiation factors. Finally, we investigated whether mutations in *EIF2B2* genes that cause VWMD perturb the formation of eIF2B bodies, SGs or P-bodies by affecting the structure or function of the eIF2B complex (respectively).

We found that eIF2B bodies are induced during acute glucose deprivation stress in yeast. Further, disease-causing mutations in eIF2B subunits had variable impacts on eIF2B bodies, SGs or P-bodies during glucose deprivation stress, supporting the idea that VWMD mutations likely cause disease through an alternative mechanism. Interestingly, we observed that some mutations in the *EIF2B2* subunit led to hypersensitivity to chronic phosphorylation of eIF2α, which is consistent with VWMD mutations altering the response to stress in a manner that might trigger increased cell death^18^.

## Results

### EIF2B bodies are induced by acute glucose deprivation stress

Previous studies on the conditions that induce eIF2B bodies to form in yeast are contradictory. It has been reported that eIF2B bodies are constitutively present in log phase cultures^5,8,19^. In contrast, other studies report that eIF2B bodies are not formed in yeast at log phase, but are induced in response to conditions of long-term nutrient starvation when the cytoplasmic pH is low^6,7^. These issues might be due to differences in experimental conditions, and/or the eIF2B protein examined. To clarify these issues and develop robust conditions to examine eIF2B body formation, we first surveyed eIF2B body formation in several contexts, and by visualizing all five subunits of eIF2B via analysis of eIF2B-GFP fusion proteins as has been previously described^5–8,19^.

We hypothesized that acute glucose deprivation could cause eIF2B body formation, as this condition causes low cytoplasmic pH, cellular energy depletion and translation repression^20–22^ as would be expected to occur during long-term starvation^23^. Indeed, a previous study showed qualitative evidence that one subunit of the eIF2 complex, eIF2α, can form fibers in yeast during glucose deprivation stress^24^. We therefore monitored the subcellular localization of all five eIF2B proteins (GCN3, GCD7, GCD1, GCD2 and GCD6) and eIF2α (SUI2) using strains from the GFP fusion collection^25^, as GFP-tagged eIF2B proteins can form/be recruited to eIF2B bodies^8^. Cultures were grown to log phase and switched to glucose -depleted or -replete medium for 30 minutes. Yeast were then concentrated through a brief (~5 sec), low speed centrifugation step and immediately imaged (without washing) under agarose pads on a cover glass using a spinning disc confocal microscope to assess localization of GFP fusion proteins to minimize any potential stress responses due to cell washing, oxygen deprivation and light.

**Figure 1:**
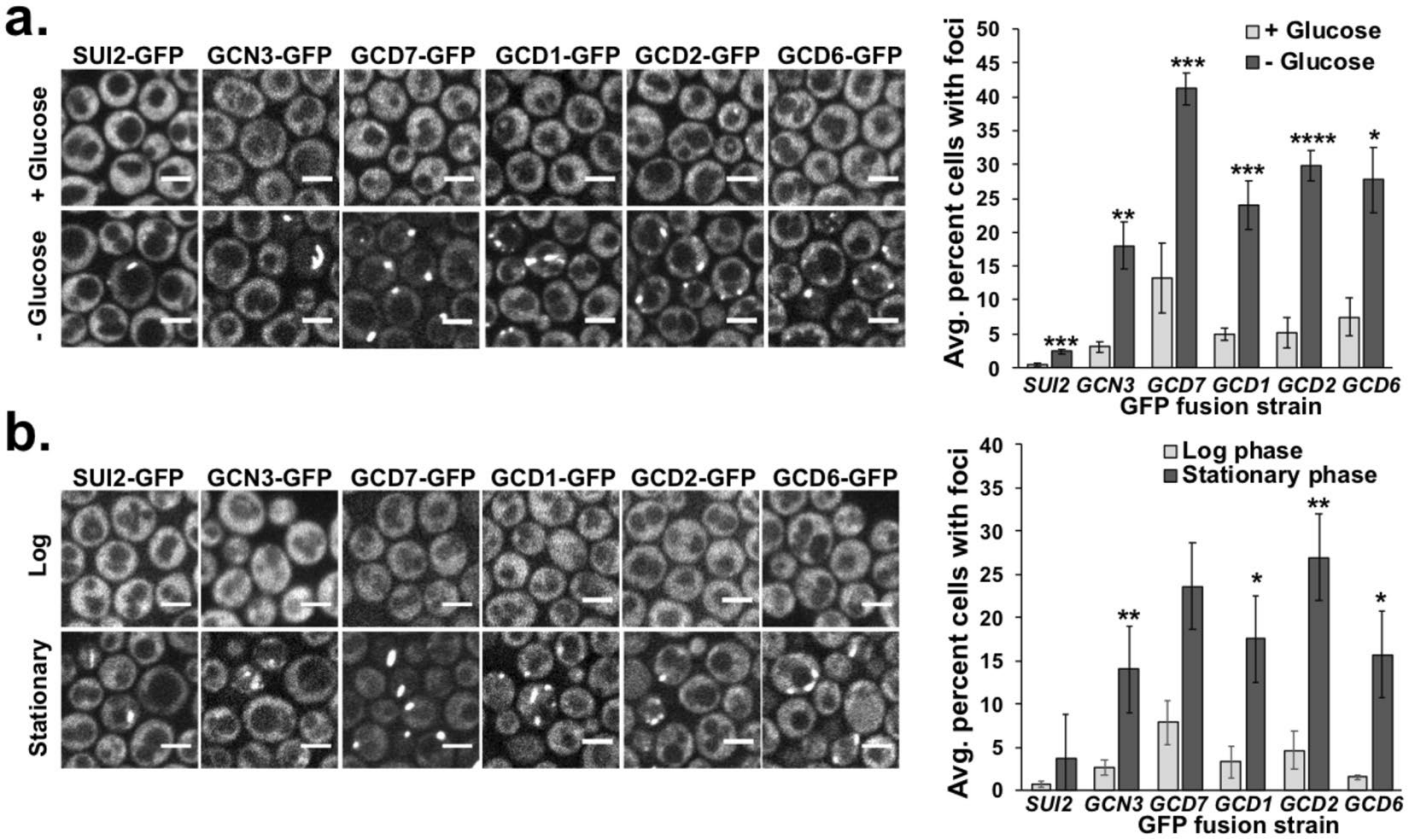
EIF2B bodies are induced by acute glucose deprivation stress and form during stationary phase. (**a**) Strains with genomic GFP tagged eIF2α (SUI2) or the five eIF2B subunits (GCN3, GCD7, GCD1, GCD2 and GCD6) were grown in complete minimal medium to log phase, then starved of glucose (“−glucose”) or placed in medium containing 2% glucose (“+glucose”) for 30 minutes. Yeast were imaged at 100x under 1% agarose pads on a spinning disc confocal microscope. Representative images of the localization of GFP tagged SUI2, GCN3, GCD7, GCD1, GCD2 and GCD6 under normal (“+glucose”) or acute glucose deprivation (“−glucose”) conditions are shown at left. Images were cropped from full sized frames of ~250–500 cells and scale bars are 5 μm. The average percent cells containing eIF2B bodies (defined as foci or fibers) from 3 independent biological replicates is shown +/− SEM at right. (**b**) Yeast grown to log or stationary phase were imaged as above. Approximately 250–500 cells were counted for each replicate and scale bars are 5 μm. The average percent cells containing eIF2B bodies from 3–5 independent experiments is shown +/− SEM at right. Student’s t-test was used to assess significance between +glucose and −glucose conditions (**a**) or log and stationary phase (**b**) with * indicating p < 0.05, **p < 0.01, ***p < 0.005 and **** is p < 0.001.

We found that eIF2B bodies were infrequently observed in unstressed conditions in all six GFP strains, and on average were only present in foci or fibers in 7% of all unstressed cells (Fig. 1a). However, a 30-minute glucose deprivation stress caused a ~5-fold increase in the number of cells (20–40% of the cells) with eIF2B bodies compared to unstressed conditions (Fig. 1a). We observed a similar increase in cells with eIF2B bodies upon glucose deprivation in a different yeast strain transformed with a plasmid containing GCD7-mRuby2 visualized under glass coverslips (Fig. S1). It is possible that the variation in the number of eIF2B bodies formed in each strain is due to effects of the GFP tag on protein localization. However, previous studies have shown there are no defects in cell growth or viability in strains with the essential *EIF2B* genes tagged with c-terminal GFP tags and have validated use of GFP fusion proteins for visualizing eIF2B bodies in cells by immunofluorescence assays^5,8^. Furthermore, glucose starvation-induced eIF2B bodies were rapidly dissolved upon glucose repletion (Video S1). These results demonstrate that eIF2B bodies are dynamically induced and dissolved in response to short-term changes in glucose availability. It is also possible that eIF2B bodies may rapidly form and dissolve in normal growth conditions, but this dynamic assembly-disassembly process could be perturbed in the absence of glucose.

In contrast to eIF2B proteins being assembled into eIF2B bodies in roughly 20–40% of cells upon glucose deprivation, eIF2α (SUI2-GFP) was only present in 2.4 +/− 0.47% cells during glucose deprivation. However, SUI2 was shown to cycle in and out of eIF2B bodies^5,19^ and our results are in-line with the idea that SUI2 is not a resident protein for eIF2B bodies, but rather dynamically associates with these aggregates, presumably through its physical interaction with the eIF2B complex.

Since eIF2B bodies were increased by acute glucose deprivation, we also compared log-phase cultures with those that had grown to stationary phase, which depletes glucose. We observed that eIF2B bodies were increased on average 6-fold over log phase cultures, similar to what we observed in acute glucose deprivation stress (Fig. 1b). In sum, these results lend further support for the notion that eIF2B bodies form when glucose is limiting but can be rapidly disassembled in response to glucose repletion.

Glucose deprivation stress causes pH to decrease in the cytoplasm^20,21^, and eIF2B body formation during long term chronic nutrient deprivation requires low cytoplasmic pH^6^. Prior studies demonstrated the reliance of eIF2B body formation on acidification of the cytoplasm by artificially reducing pH using protonophores and/or energy depleting compounds including 2-deoxyglucose, sodium azide and antimycin A to suppress glycolysis and oxidative phosphorylation^6,7^. To extend these studies, we used the genetic pH sensor pHuji^26^ to take an unbiased approach to assessing whether eIF2B bodies form in single cells upon cytoplasmic acidification during glucose deprivation stress. pHuji was used to measure cytoplasmic pH in yeast because it enables simultaneous evaluation of both pH and the localization of GFP fusion proteins. As expected, glucose deprivation stress caused a reduction in cytoplasmic pH in most cells, as the intensity of the pH sensor pHuji was significantly reduced by ~25% in GCD1-GFP tagged cells after 30 minutes of glucose deprivation stress (Fig. S2).

Quantification of the percent of cells with eIF2B bodies and the total pHuji intensity in the GCD1-GFP strain over a 15-minute time-lapse image series after glucose repletion showed that eIF2B body disassembly corresponds with a rapid increase in cytoplasmic pH (Fig. S2 and Video S2). Therefore, a dynamic reduction in cytoplasmic pH upon glucose deprivation is associated with eIF2B body formation.

### EIF2B bodies assemble and disassemble independently of stress granules

**Figure 2:**
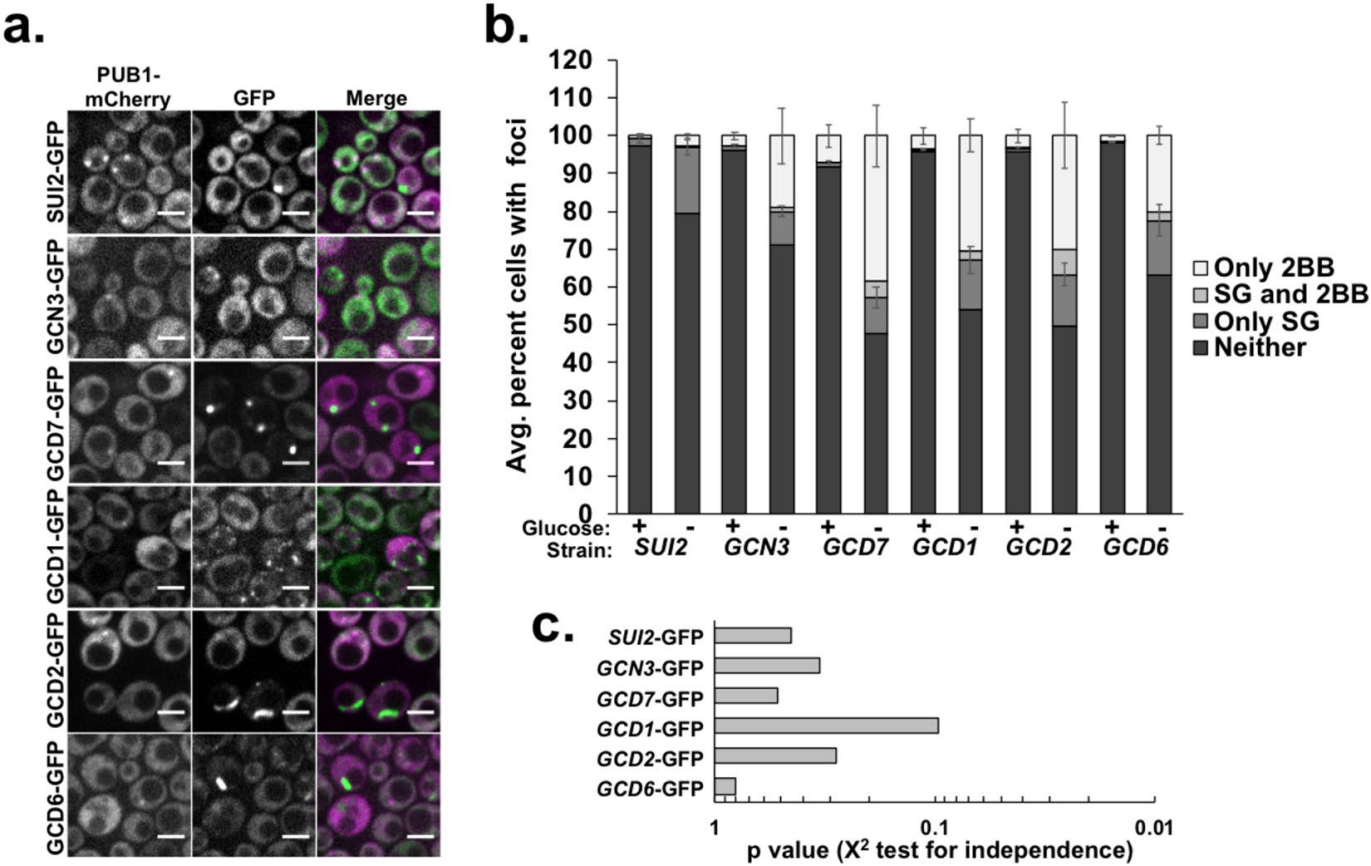
EIF2B bodies arise independently from stress granules during acute glucose deprivation. The indicated GFP fusion strains were transformed with a plasmid encoding an mCherry tagged stress granule protein (PUB1-mCherry), grown to log phase and switched to medium with or without glucose for 30 minutes. Yeast were imaged under 1% agarose pads at 100x on a confocal spinning disc microscope. (**a**) Representative images of stress granules (marked by PUB1-mCherry) and eIF2B bodies in GFP fusion strains after a 30-minute glucose deprivation stress. Scale bars are 5 μm. (**b**) Quantification of the number of cells with stress granules (“SG”) and/or eIF2B bodies (“2BB”) at log phase in the presence or absence of glucose (30 minutes), with the average +/− standard error of the mean from 200–400 cells per replicate, from 3–6 biological replicates shown. (**c**) Graphical representation of p-values determined using a chi-squared test for independence to assess whether stress granules and eIF2B bodies were present in the same cells or occurred independently of one another.

Glucose deprivation stress also induces the formation of SGs: RNA-protein aggregates that are enriched in translation factors. However, although SGs and eIF2B bodies are both induced by long-term starvation, growth and glucose deprivation, and both foci harbor translation factors, the relationship between SGs and eIF2B bodies has not been evaluated to date. To determine if eIF2B bodies are related temporally (i.e. if they are induced at the same time during stress) or physically interact with SGs, we transformed *EIF2B*- and *SUI2*- GFP fusion strains with a plasmid encoding the stress granule protein PUB1 tagged with mCherry^24^. The number of cells that had eIF2B bodies, SGs, or both in each strain was then assessed following glucose deprivation stress or in unstressed cells at log phase (Fig. 2).

We observed that while both SGs and eIF2B bodies were induced upon glucose deprivation, cells appeared to form either eIF2B bodies or SGs and infrequently contained both. Indeed, as determined using a chi squared test for independence, the proportion of cells that formed both eIF2B bodies and SGs was as expected if they arose in the same cell by chance alone (Fig. 2c). In those cells that contained both eIF2B bodies and SGs, these assemblies could either co-localize, be adjacent to one another, or be localized far away from one another (Fig. S3). These results suggest that eIF2B bodies and SGs are independent molecular assemblies.

We observed that more cells contained eIF2B bodies than SGs after a 30-minute glucose deprivation stress (Fig. 2b) and the percent of cells with GCD1-GFP and GCD7-GFP foci was significantly higher than cells with SGs (p ≤ 0.05, Student’s t-test). These findings imply that the kinetics of stress granule and eIF2B body assembly may be different and that they may be regulated by distinct biochemical processes. EIF2B bodies may therefore form more rapidly (and/or be more stable) upon glucose deprivation stress than SGs.

**Figure 3:**
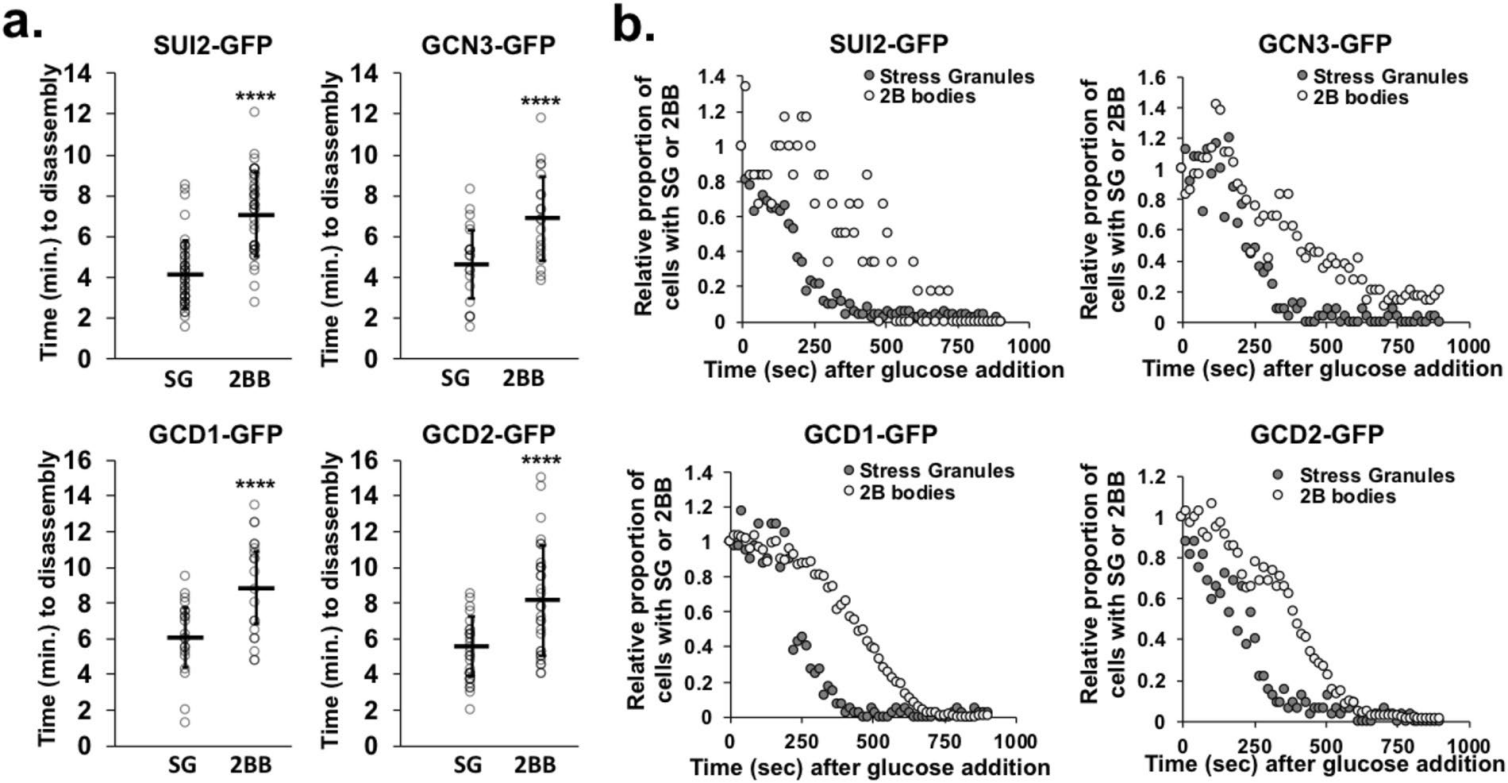
Stress granules induced by glucose deprivation stress dissolve before eIF2B bodies upon glucose repletion. Strains with endogenously GFP tagged *SUI2*, *GCN3*, *GCD1* and *GCD2* were transformed with a stress granule marker plasmid PUB1-mCherry and imaged under a 1% agarose pad on a spinning disc confocal microscope (100×). A solution of 10% glucose was added at time 0 and cells were imaged every 15 seconds for 15 minutes. (**a**) Graphs show the time (minutes) until stress granules (“SG”) and eIF2B bodies (“2B bodies” or “2BB”) dissolved in individual cells after glucose repletion. Each dot represents the time all foci dissolved in a single cell. The average +/− standard deviation is plotted overlaying individual data points and represents 20–40 individual cells from 2–4 independent experiments. Student’s t-test was used to assess significance with **** denoting p ≤ 0.001. (**b**) Quantification of the relative number of eIF2B bodies (light gray dots) and stress granules (dark gray dots) in the population of cells in each frame with frame 1 set to 1.0 in a time-lapse image (~300–400 cells per frame) following glucose repletion.

The kinetics of stress granule and eIF2B body disassembly upon glucose repletion were also different (Fig. 3), further supporting the idea that these assemblies are regulated through distinct processes. We tracked the disassembly of these cytoplasmic aggregates during glucose repletion by applying a solution of 10% glucose to the agarose pads overlaying each sample on the microscope stage such that the final glucose concentration cells are exposed to should be ~2%. Images were then obtained every 15 seconds for 15 minutes. We tracked the time post-glucose addition at which all eIF2B bodies or all SGs dissolved in individual cells (with 20–40 individual cells evaluated per strain) (Fig. 3a). Interestingly, SGs dissolved almost immediately upon cellular uptake of glucose following diffusion of the fresh medium through the agarose pad as determined by monitoring bulk changes in cytoplasmic pH using a green genetic pH sensor super ecliptic pHluorin (Fig. S4)^27^. On average, SGs dissolved at 5.1 +/− 0.9 minutes (average +/− SD), significantly faster (p = 0.003124, Student’s t-test) upon glucose repletion than eIF2B bodies, which dissolved 7.8 +/− 0.9 minutes post-glucose repletion in individual cells. Evidence that eIF2B bodies disassemble slower than SGs in the four strains tested (*SUI2*-GFP, *GCN3*-GFP, *GCD1*-GFP and *GCD2*-GFP) came from analyzing the full population (Fig. 3b). Stress granules consistently disappeared from the population almost twice as fast as eIF2B bodies, with the time during recovery at which the population had ~50% the initial level of SGs or eIF2B bodies upon glucose deprivation stress being ~250 or ~500 seconds (respectively) post-glucose repletion (Fig. 3b). Taken together, our findings demonstrate that although eIF2B bodies and SGs form under similar conditions, they are likely regulated through distinct pathways, have differential sensitivity to translation and energy status in the cell, and/or have distinct biophysical properties that contribute to their differential disassembly kinetics.

### Mutations causative of VWMD do not consistently impact eIF2B body formation

Mutations in *EIF2B* genes cause a fatal leukodystrophy called Vanishing White Matter disease, wherein white matter loss can be triggered or worsened by stress, trauma or illness through an unknown mechanism^13^. Because many mutations in *EIF2B* genes cause reduced guanine exchange factor activity and therefore likely suppress translation initiation in unstressed and stressed conditions, we hypothesized that cells harboring VWMD mutations may alter translation in unpredicted manners leading to elevated stress-induced macromolecular assemblies. Further, a previous study identified mutations in GCN3 (R148K and T41K) that abrogate eIF2B body formation in butanol-resistant suppressor strains^19^, demonstrating that specific mutations in eIF2B genes can impact eIF2B bodies. We therefore determined if the formation of eIF2B bodies, SGs and P-bodies were altered in a panel of thirteen yeast strains harboring one copy of VWMD mutant or wild-type *GCD7* (*EIF2B2*) or *GCD6* (*EIF2B5*)^28^. *S*. *cerevisiae* was chosen as a model system because EIF2 and EIF2B genes are highly conserved, there is a paucity of available human patient-derived cell lines, using yeast mitigates the effects of inter-individual genetic variability and because a prior study analyzed translation activity in these strains, allowing direct comparison to observed changes in stress-induced cytoplasmic granules. Although these strains lack the eIF2α kinase GCN2, glucose deprivation stress causes translational repression through a p-eIF2α - independent mechanism downstream of eIF2B in the translation initiation pathway^22^ and we therefore reasoned they would be an appropriate model for studying eIF2α - independent translational repression.

**Figure 4:**
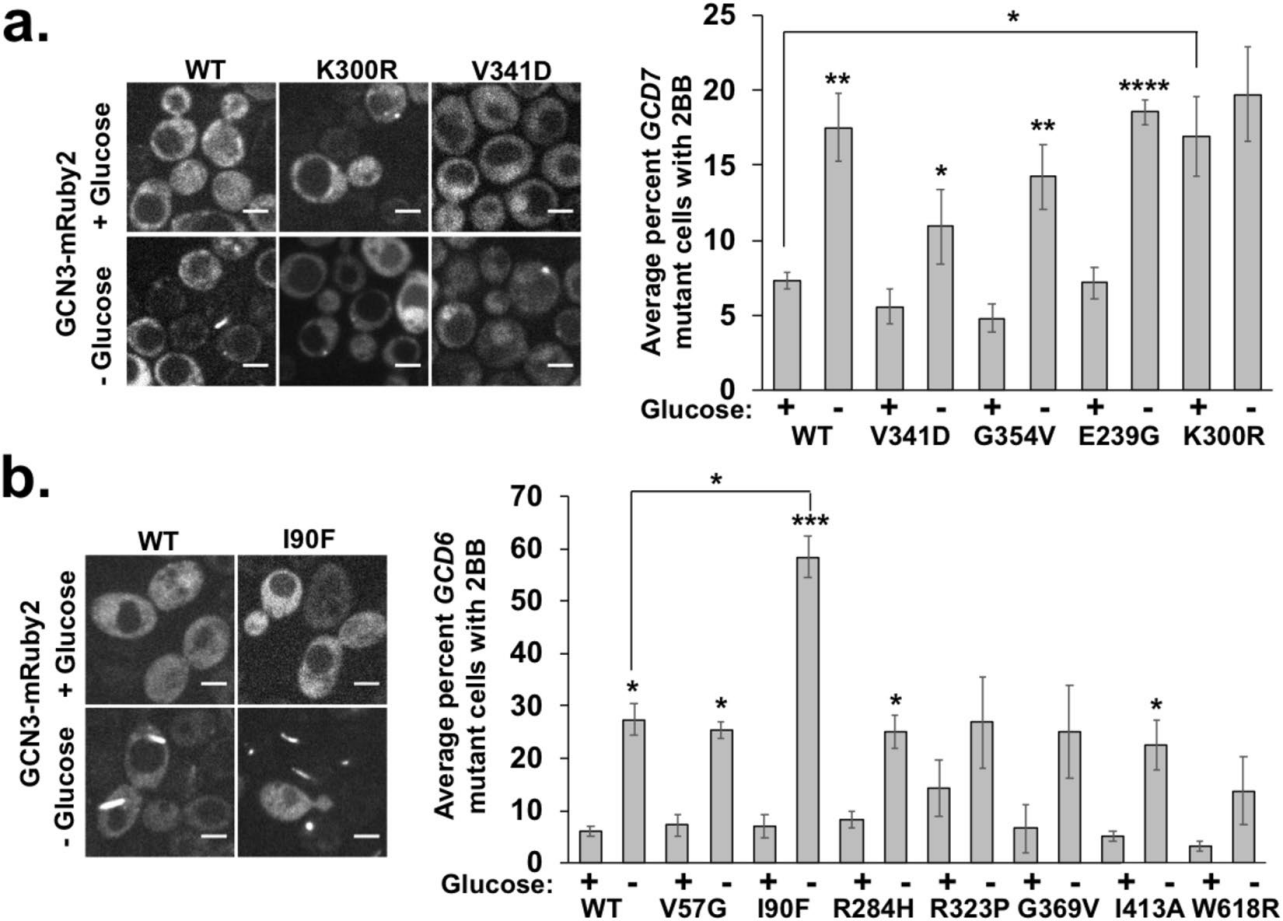
Certain mutations in *EIF2B2* or *EIF2B5* causative of VWMD cause dysregulation of eIF2B bodies in yeast. A panel of yeast harboring VWMD mutations in *GCD7* or *GCD6*^28^ were transformed with a plasmid encoding GCN3 tagged with mRuby2 to visualize eIF2B bodies. Yeast were grown to log phase and incubated for 30 minutes under glucose replete or depleted conditions. Cultures were imaged under 1% agarose pads on a spinning disc confocal microscope at 100x magnification. (**a**) Changes in the percent cells with eIF2B bodies (“2BB”) in yeast expressing wild-type or VWMD mutant *GCD7*. Representative images are shown of the wild-type *GCD7*, *GCD7* K300R and *GCD7* V341D strains at left with 5 μm scale bars. Results presented at right are the average of 3–5 independent biological replicates +/− SEM, with 200–500 cells counted per replicate. (**b**) Relative percent cells with eIF2B bodies (“2BB”) in *GCD6* wild-type or mutant strains. Representative images are shown of the wild-type *GCD6* and *GCD6* I90F strains at left with 5 μm scale bars. Results at right are the average of 2–3 biological replicates with 200–500 cells counted for each replicate +/− SEM. Student’s t-test was used to assess significance with * indicating p ≤ 0.05, **p ≤ 0.01, ***p ≤ 0.005 and ****p ≤ 0.001.

First, we assessed the induction of eIF2B bodies in *GCD6* and *GCD7* mutants by visualizing the localization of a GCN3-mRuby2 fusion protein encoded on a CEN plasmid and driven by its own promoter in these cells. As expected, the abundance of GCN3-mRuby2-containing eIF2B bodies increased 2-5-fold upon glucose deprivation stress in wild-type yeast (Fig. 4) and co-localized with GCD2-GFP in eIF2B bodies (Fig. S5). Therefore, the induction of eIF2B bodies under glucose deprivation stress is not strain-specific and the GCN3-mRuby2 fusion protein is suitable for labeling eIF2B bodies.

In general, most VWMD alleles in *GCD7* or *GCD6* did not alter eIF2B body formation. Specifically, of the four *GCD7* and seven *GCD6* mutant strains examined, only three mutants affected eIF2B body abundance in stressed or unstressed conditions compared to wild-type cells. One allele, *GCD7* K300R (analogous to *EIF2B2* K273R), led to an increase in eIF2B bodies in unstressed cells (15 +/− 4.7%) compared to wild-type cells (7.5 +/− 0.95%), but failed to increase eIF2B bodies further upon glucose depletion (Fig. 4a). A qualitative assessment of eIF2B body morphology revealed the K300R strain had smaller, more punctate eIF2B bodies than long fibril-like aggregates compared to those found in wild-type cells (Fig. 4a). Similarly, the *GCD7* V341D strain (analogous to *EIF2B2* V316D) formed eIF2B bodies that were invariably round and punctate instead of fiber-like as observed in wild-type cells, although they were at similar levels as in wild-type cells and could be induced by glucose deprivation stress (Fig. 4a). One allele strongly increased eIF2B body induction with stress, where almost twice as many cells formed eIF2B bodies in the *GCD6* I90F strain (analogous to *EIF2B5* L106F) compared to wild-type cells (Fig. 4b). This suggests that this mutation either sensitizes cells to induce eIF2B bodies or stabilizes the structure of the eIF2B body thereby increasing their abundance. Since we did not observe a consistent effect of VWMD mutations on eIF2B body induction or morphology, we conclude that eIF2B body dysregulation does not likely play a major role in VWMD pathogenesis.

**Figure 5:**
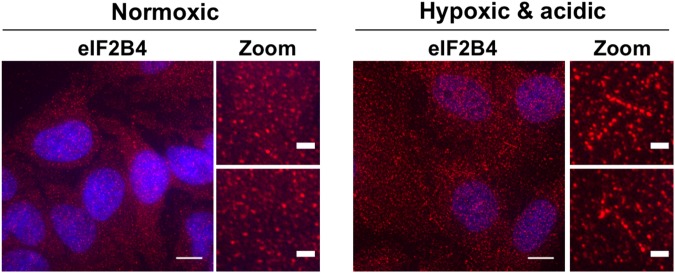
EIF2B4 can localize to foci and fibers in HeLa cells. Endogenous eIF2B4 protein (red) was detected by indirect immunofluorescence microscopy in cells under normoxic (left) or hypoxic and acidic conditions (right, 24 hours). Nuclei were stained with DAPI (blue). Cells were imaged with 100x objectives with scale bars indicating 10 μm or 2 μm (magnified image panels). Results represent at least three independent experiments.

We also evaluated whether eIF2B bodies could form in human tissue culture cells. In normal growth conditions, using antibodies against the eIF2B subunit, eIF2B4, we observed eIF2B was slightly punctate but generally distributed throughout the cytosol with no fiber-like assemblies analogous to yeast eIF2B bodies (Fig. 5, left panel). This distributed localization of eIF2B is consistent with a recent survey of subcellular localization of the human proteome that revealed eIF2B proteins are diffusely cytoplasmic in normal cell culture growth conditions^29^.

To evaluate whether eIF2B bodies could form under conditions that acidify the cytoplasm, we examined the localization of eIF2B4 in HeLa cells under normal growth conditions and hypoxic, acidic (pH ~6) conditions by indirect immunofluorescence microscopy. We observed eIF2B4 was present in rod-like filament structures after cells were exposed to a hypoxic, slightly acidic (~0.1% O_2_ and 20% CO_2_) environment for 24 hours (Fig. 5, right panel). This demonstrates that some fiber- like assemblies of eIF2B can form under hypoxic conditions in mammalian cells, although whether these are strictly analogous to eIF2B bodies seen in yeast or rather represent an instance of dynamic recruitment of eIF2B4 proteins to a constitutively present filament will require additional work beyond the scope of the current study.

### VWMD mutations can cause increased P-body levels, but generally do not alter stress granules

**Figure 6:**
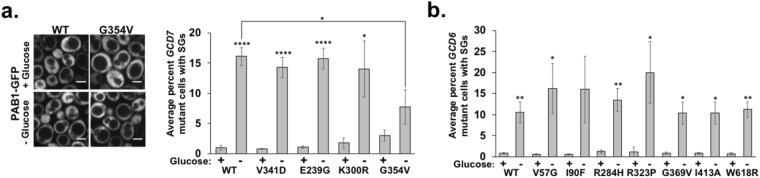
Stress granule induction by glucose deprivation stress is generally unperturbed by mutations in *GCD7* (*EIF2B2*) or *GCD6* (*EIF2B5*) that cause VWMD. Yeast strains harboring *GCD7* or *GCD6* mutations were transformed with a plasmid encoding PAB1-GFP and EDC3-mCherry to mark stress granules and P-bodies (respectively; P-bodies shown in Fig. 7). Yeast were imaged under 1% agarose pads on a spinning disc confocal microscope at 100x magnification. The number of cells with stress granules (“SGs”) was counted in log phase and in cells exposed to 30 minutes of glucose starvation. (**a**) Stress granule abundance in *GCD7* mutant strains. At left, representative images showing PAB1-GFP localization in the wild-type *GCD7* strain or the *GCD7* G354V strain under glucose replete or depleted conditions. Scale bars indicate 5 microns. At right, the average +/− SEM of the percent cells with stress granules from 3 independent experiments. (**b**) The average +/− SEM of the percent of cells with stress granules in *GCD6* mutant strains. Results are from 2–3 biological replicates, with ~100–900 cells counted per replicate. Student’s t-test was used to assess significance, with * indicating p ≤ 0.05, **p ≤ 0.01 and ****p ≤ 0.001.

**Figure 7:**
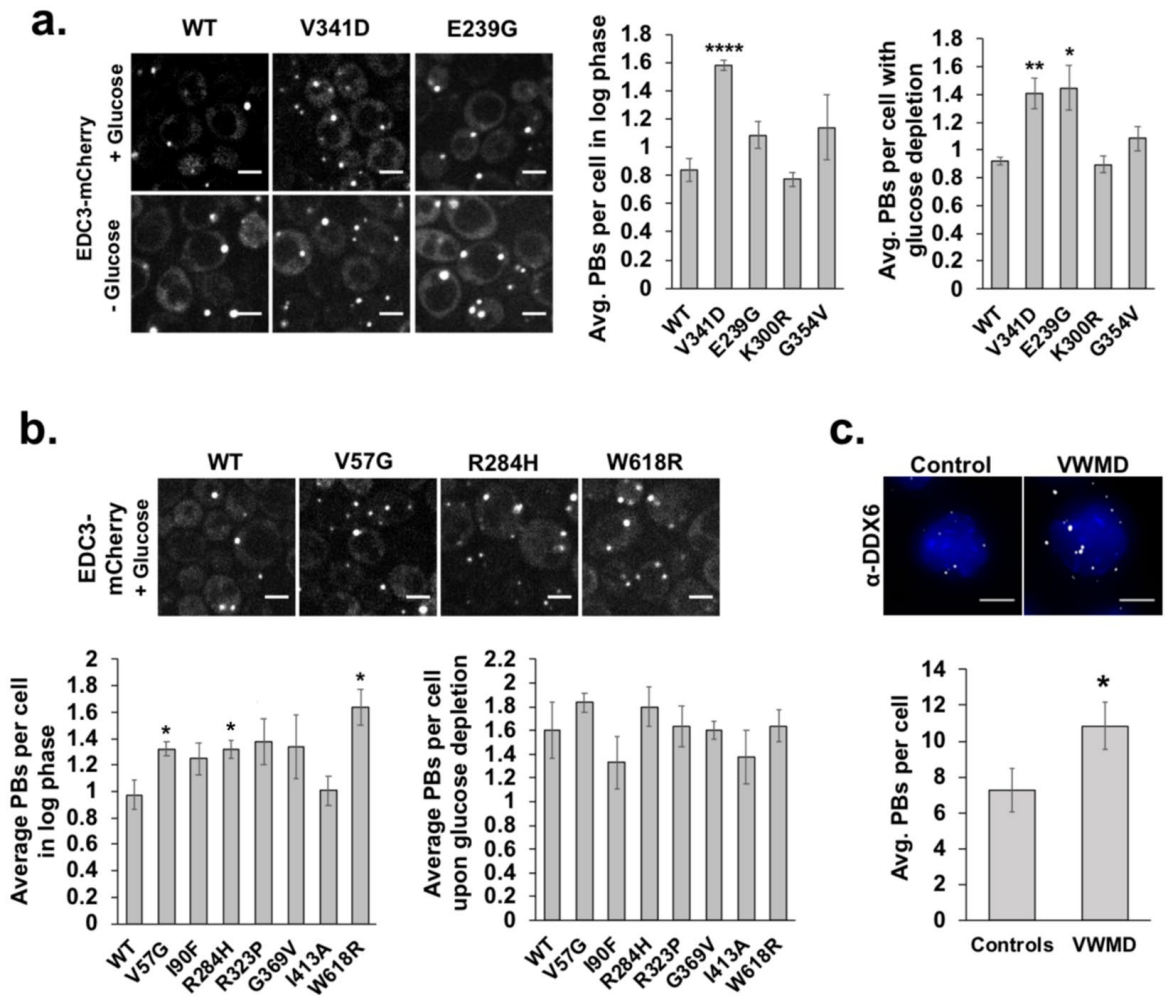
Yeast encoding certain mutations in *GCD7* (*EIF2B2*) or *GCD6* (*EIF2B5*) analogous to VWMD patient mutations and immortalized lymphoblasts derived from two VWMD patients have elevated P-bodies. Yeast were transformed with a plasmid encoding EDC3-mCherry (a P-body marker) and PAB1-GFP (a stress granule marker, data shown in Fig. 6), grown to log phase and kept in glucose replete or depleted medium for 30 minutes prior to imaging on a spinning disc confocal microscope at 100x magnification. The number of P-bodies (“PBs”) per cell in *GCD7* strains (**a**) or *GCD6* strains (**b**) was determined in ~100–150 cells per biological replicate and graphs depict the average +/− SEM from 2–4 biological replicates. Representative images of strains with elevated P-bodies under glucose replete or depleted conditions (**a**) or under log phase glucose replete conditions alone (**b**) are shown. Scale bars are 5 μm. (**c**) P-bodies were detected in immortalized lymphoblasts derived from two health controls “Controls” or two siblings with VWMD “VWMD” harboring *EIF2B2*^*E304X*^ mutations by immunofluorescence microscopy to detect the P-body protein DDX6. Shown (top) are max projections of Z-stacks of representative cells imaged at 100x (scale bar is 5 μm). Graphed below is the average +/− SEM of the number of P-bodies per cell from two different control and two different VWMD cell lines from four independent experiments. Student’s t-test was used to assess significance, with * indicating p ≤ 0.05 and ****p ≤ 0.001.

We also assessed the effect of *GCD6* and *GCD7* mutations that cause VWMD on SGs and P-bodies. Strains harboring wild-type or mutant *GCD6* or *GCD7* plasmids were transformed with a plasmid encoding a fluorescent protein-tagged SG (PAB1-GFP) and P-body (EDC3-mCherry) protein^24^. The number of cells with SGs (Fig. 6) and the number of P-bodies per cell (Fig. 7) in unstressed and glucose deprivation-stressed cells for each strain was determined as above.

Surprisingly, although eIF2B is a general translation factor required for ternary complex formation, there were no obvious defects in SG induction upon glucose deprivation stress or in unstressed conditions in cells harboring VWMD mutations in *GCD6* or *GCD7* (Fig. 6). There was a slight but significant reduction in the number of cells with SGs during glucose deprivation stress in the *GCD7* G354V mutant (Fig. 6a). We conclude that SGs are not constitutively present or generally affected during glucose deprivation stress by the mutations analyzed in the yeast homologs of *EIF2B2* or *EIF2B5* known to cause VWMD.

In contrast, we found that several *GCD6* and *GCD7* mutants affected P-bodies. First, the number of constitutive P-bodies per cell was significantly elevated in the *GCD7* V341D, *GCD6* V57G, *GCD6* R284H and *GCD6* W618R strains compared to wild-type cells (Fig. 7a,b). Since P-bodies are increased by defects in translation initiation^1^, the simplest interpretation of this observation is that the VWMD mutations cause a slight reduction in the translation of some mRNAs, and those untranslated mRNAs are accumulating to some extent in P-bodies. Similarly, we observed that during glucose deprivation stress *GCD7* V341D and *GCD7* E239G strains had increased levels of P-bodies (Fig. 7a), which could also be due to increased translation repression under these conditions due to these alleles. Since the size of P-bodies reflects the association of mRNAs with the mRNA degradation machinery, an increase in P-bodies due to certain eIF2B mutations implies that these mutations alter the degradation rates of some mRNAs.

To examine if VWMD mutations could also affect P-bodies in human cells in culture, we examined immortalized lymphoblasts derived from two siblings with VWMD harboring *EIF2B2*^*E304X*^ mutations. We observed that P-bodies were significantly increased in VWMD patient cells under normal growth conditions compared to those from age and sex matched healthy controls (Fig. 7c). Thus, VWMD mutations can cause an increase in P-bodies in both yeast and mammalian cells in culture.

### VWMD-associated mutations in GCD7 sensitize cells to chronic stress

We next examined how yeast strains with a single copy of mutant *GCD6* or *GCD7*^28^ tolerated chronic stress conditions by monitoring their growth in the presence of a constitutively active form of the GCN2 kinase, which normally phosphorylates eIF2α upon amino acid starvation, but when constitutively active causes chronic eIF2α phosphorylation^30^ and constitutive SGs^24^. We evaluated yeast growth at the optimal temperature of 30 °C and at sub-optimal temperatures of 25 °C or 37 °C to compare wild-type and mutant strains under conditions that can alter growth rates. The *GCD6* strains grew poorly on solid medium following transformation with empty vector or plasmids encoding wild-type or constitutively active GCN2, and we therefore did not assess their relative growth.

**Figure 8:**
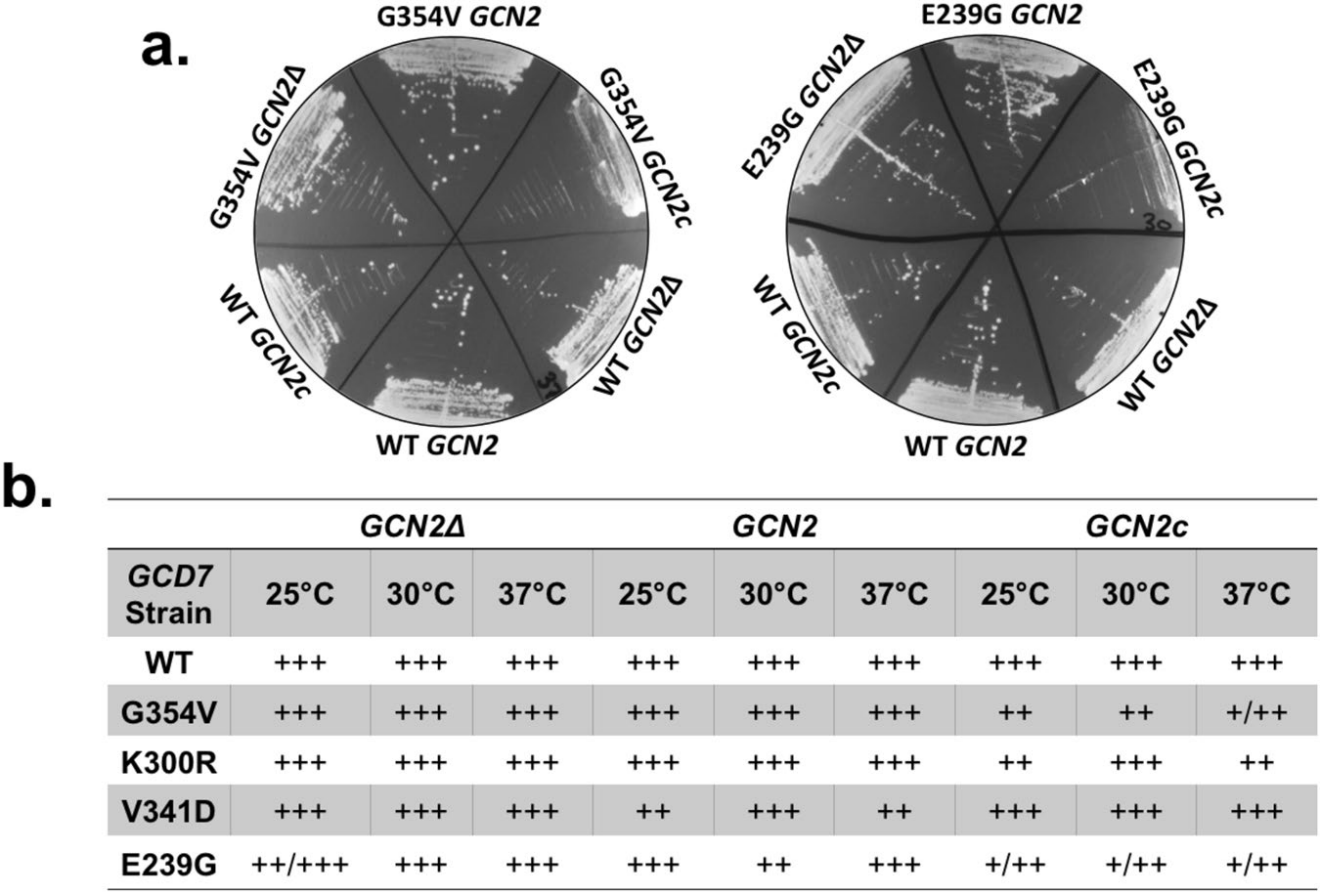
VWMD-causing mutations in *GCD7* hyper-sensitize yeast cells to chronic stress. (**a**) Representative images showing differential growth of yeast harboring G354V (grown at 37 °C) or E239G (grown at 30 °C) mutations in *GCD7* (the yeast homolog of *EIF2B2*) in the absence of *GCN2* (“*GCN2Δ*”), the presence of *GCN2* (“*GCN2*”) or a constitutively active allele of *GCN2* (“*GCN2c*”). (**b**) Summary of relative growth of mutant *GCD7* strains compared to wild-type yeast grown at 25 °C, 30 °C and 37 °C from 2–3 independent clones. “+” indicates poor growth, “++” indicates intermediate growth and “+++” indicates strong growth.

Interestingly, we observed that *GCD7* strains expressing the G354V, K300R or E239G variants grew similar to wild-type yeast in the absence of *GCN2*, but in the presence of wild-type or a constitutive *GCN2* allele (“GCN2c”), all VWMD-mutant strains showed reduced growth at some temperature. Because GCN2 is the only kinase in yeast that phosphorylates eIF2α and the constitutively active GCN2 allele causes eIF2α phosphorylation, our results suggest these VWMD alleles made cells sensitive to phosphorylation of eIF2α (Fig. 8). These results are in-line with our previously observations that VWMD patient lymphoblasts with *EIF2B2*^*E304X*^ mutations are hyper-sensitive to chronic, severe ER stress^18^. Therefore, mutations in the *GCD7* subunit of the eIF2B complex can confer sensitivity to chronic stresses that activate the p-eIF2α signaling pathway and the integrated stress response.

## Discussion

We describe cellular conditions that affect eIF2B body formation, which will be important for understanding the formation and function of eIF2B bodies. In contrast to previous reports that eIF2B bodies are abundantly formed in normal conditions^5,8^, our results indicate that eIF2B bodies occur in less than 10% of cells under normal conditions at log phase, but are rapidly induced by glucose deprivation stress and during stationary phase when glucose is limiting. These results agree with two recent studies that demonstrate eIF2B bodies are induced under conditions of long-term nutrient deprivation stress^6,7^, which we would now extend to a short term (30-minute) glucose deprivation. Our results also highlight that eIF2B body formation is independent of stress granule formation and suggest that eIF2B bodies are dynamic assemblies that can be rapidly assembled and disassembled in the absence or presence of glucose. The overall implication of the results of this study are that eIF2B bodies form under conditions of translational repression and are not part of the constitutive organization of translation factors in the cytoplasm. In accordance with this implication, previous studies have demonstrated that trafficking of eIF2α through eIF2B bodies is slowed or reduced when translation is repressed (by acute butanol stress^19^, amino acid deprivation^5^, or overexpression of eIF5 mimic protein 1 suppresses of eIF2 activity^31^). Therefore, the current study supports the notion that eIF2B bodies might serve as an indicator of translation activity in the cell as has been previously suggested^5,19^, although future studies should directly test this hypothesis.

In support of the observation that yeast cells do not normally harbor eIF2B bodies at log phase, we provide evidence that eIF2B bodies do not normally form in human tissue culture cells but may assemble into fiber-like structures under severe hypoxic and acidic conditions. Hypoxia and acidosis can occur in solid tumors and ischemic tissues^32–36^, and re-localization of eIF2B proteins to distinct cytoplasmic structures could play a role in cell survival in these contexts. Because hypoxia causes translational repression in mammalian cells^37^, these results further support the idea that eIF2B bodies are induced when translation is suppressed. Future work should seek to characterize these structures in mammalian cells and determine what other conditions may induce their formation.

In this work, we also examined how mutations in the eIF2B subunits that cause VWMD affect the formation of eIF2B bodies, SGs, and P-bodies. Although 8 out of 11 strains tested had significantly altered eIF2B bodies, SGs or P-bodies, only one of these strains (*GCD7* V341D) affected more than one of these macromolecular assemblies (Table 1). The *GCD7* V341D mutant had increased constitutive P-bodies and smaller, punctate eIF2B bodies compared to wild-type cells (Fig. 4a). Interestingly, this mutant grows more slowly and has reduced translation activity as was measured by polysome profiling in a previous study^28^ consistent with the idea that repressed translation initiation activity promotes P-body formation^1^.

**Table 1:** Summary of significantly different eIF2B body, stress granule and P-body phenotypes observed and location of mutations in the three-dimensional GCD7 or GCD6 protein structure^38^.

Strain	Location in protein^38^	2B body phenotype	Stress granule phenotype	P-body phenotype
*GCD7* V341D	Core	Round/punctate morphology	N/A	Increased (constitutive)
*GCD7* G354V	Subunit interface	N/A	Reduced during stress	N/A
*GCD7* E239G	Subunit interface	N/A	N/A	Increased (stress-induced)
*GCD7* K300R	Core/interdomain pocket	Increased (constitutive); not stress-induced	N/A	N/A
*GCD6* V57G	Core	N/A	N/A	Increased (constitutive)
*GCD6* I90F	Core	Increased (stress induced)	N/A	N/A
*GCD6* R284H	Core	N/A	N/A	Increased (constitutive)
*GCD6* R323P	Subunit interface	N/A	N/A	N/A
*GCD6* G369V	Exposed	N/A	N/A	N/A
*GCD6* I413A	Core	N/A	N/A	N/A
*GCD6* W618R	HEAT domain	N/A	N/A	Increased (constitutive)

Two strains, *GCD7* K300R and *GCD6* I90F, only affected eIF2B bodies (Table 1). The *GCD7* K300R strain had elevated constitutive eIF2B body levels but no change in constitutive P-bodies or SGs, suggesting that the observed increase in eIF2B bodies could be due to a very low level of translational suppression or an alteration in the structure of GCD7 that could promote eIF2B body formation. In contrast, there was no difference in the number of eIF2B bodies at log phase in *GCD7* I90F mutant cells compared to wild-type cells, but under glucose starvation conditions the I90F mutant had ~2x the number of eIF2B bodies compared to wild-type cells. This mutation could stabilize the structure of eIF2B bodies or cause a reduction in translation rates that might sensitize the cell to acute stress and facilitate eIF2B body formation more rapidly than in wild-type cells. On average, the number of P-bodies per cell were elevated in unstressed GCD6 I90F mutants compared to wild-type (1.25 +/− 0.21 vs 0.98 +/− 0.19) but this was not a significant difference (p = 0.084471). Furthermore, constitutive SGs were not elevated compared to wild-type cells, with less than 1% cells having SGs in both conditions (I90F: 0.49 +/− 0.26% versus WT: 0.79 +/− 0.42%). Therefore, both the *GCD7* K300R and the I90F mutations could specifically affect the structure of eIF2B bodies or their regulation without conferring a significant defect in translation activity or other stress-induced macromolecular assemblies.

A recent study^38^ determined the crystal structure of the heteropentameric *Schizosaccharomyces pombe* eIF2B complex. Because eIF2B is highly conserved^39^, the authors were able to evaluate the position of many known VWMD mutations in subunits of the eIF2B complex including those in the strains we evaluated (summarized in Table 1). We speculated that mutants that perturbed eIF2B body formation could be located at the surface of the holocomplex and could affect the assembly of multimers of eIF2B. However, the *GCD7* V341D, *GCD7* K300R and *GCD6* I90F mutations that were shown to affect eIF2B bodies were predicted to cause amino acid substitutions in the core of GCD7 and GCD6 proteins^38^ (Table 1). Because eIF2B activity is a function of the abundance of individual subunits, holocomplex formation/stability, interactions that allow reversible docking of eIF2 with the eIF2B complex, enzymatic guanine exchange factor activity^40^ and direct phosphorylation, amino acid substitutions due to missense mutations could impact any one of these and still theoretically impart a functional defect in eIF2B activity. We conclude that although VWMD mutations can impact stress-associated cytoplasmic assemblies, the lack of a common defect in these complexes negates their possible role as an underlying common aspect of VWMD pathogenesis.

P-bodies were the most commonly perturbed cytoplasmic assembly in VWMD mutant cells, often being increased under unstressed, normal growth conditions in both yeast and human cells. Increased levels of P-bodies in the absence of constitutive SGs could result from a low-level increase in non-translating mRNAs in the cytoplasm that are shuttled to P-bodies for storage and degradation. It is possible that mutants that have slightly repressed basal translation activity could promote P-body assembly through this mechanism, and subtle changes in translation activity in these mutants may have been previously missed as they were measured by polysome profiling^28^ or ^35^S metabolic labeling^18^, less sensitive read-outs of translation status in the cell.

Therefore, VWMD-causing mutations in *EIF2B2* and *EIF2B5* do not generally cause a consistent defect in P-bodies, SGs or eIF2B bodies under unstressed conditions or in response to stresses that cause translational suppression in the absence of eIF2α phosphorylation. Although our results are consistent with the highly variable nature of VWMD, we conclude that there is likely a different common mechanism underlying VWMD pathogenesis. In-line with this conclusion, we demonstrated that VWMD mutations in *GCD7* sensitize cells to a chronic stress condition that activates eIF2α phosphorylation (Fig. 8). This is consistent with the observation that certain mutations in *EIF2B2* in lymphoblasts from VWMD patients cause hyper-sensitivity to chronic ER stress^18^. Taken together with our recent study demonstrating VWMD patient cell lines hyper-suppress translation during acute stresses that activate eIF2α phosphorylation, we conclude that it is likely that mutations in *EIF2B* genes causative of VWMD primarily impact the cellular response to stress through the p-eIF2α pathway and not through alterations in stress induced assemblies independent of this translation regulation mode.

## Methods

### Yeast growth, glucose deprivation and imaging

*S*. *cerevisiae* was grown in complete or selective minimal medium with 2% glucose at 30 °C with shaking overnight, diluted and grown to log phase (OD_600_ of 0.2–0.8) for all experiments. For glucose deprivation stress, each culture was divided into two fractions, cells were pelleted briefly (~5 sec) at low speed and washed once with medium containing or lacking 2% glucose. Cells were re-suspended in medium ±2% glucose and incubated with aeration at 30 °C for 30 minutes. EIF2B body induction during stationary phase (OD_600_ of >1.0) was assessed in cells expressing the PUB1-mCherry SG marker. Cells were briefly pelleted and sandwiched between a 1% agarose LE (Light Labs) pad made with PBS and a glass-bottom dish to image. The subcellular localization of GFP-, mRuby2- and mCherry- fusion proteins were monitored at 100x on a single plane using a spinning disc confocal Nikon Ti-E microscope with an Andor 888 Ultra EMCCD camera (BioFrontiers Advanced Light Microscopy Core). ImageJ^41^ was used to count cells and foci.

### Yeast growth assays

Wild-type or mutant *GCD6* or *GCD7* (Table 2) yeast were transformed^42^ with an empty vector (pRS416), a plasmid encoding wild-type GCN2 (kindly provided by Dr. Graham Pavitt)^43^ or the constitutively active GCN2, GCN2c-515^43^ and selected on medium lacking uracil and leucine. Growth of 2–3 colonies per strain was assessed by streaking on selective medium and incubating at 25 °C, 30 °C or 37 °C for two days. Growth was scored based on size and abundance of colonies relative to wild-type GCD7 cultures streaked on the same plate, with “+++” indicating strong growth and/or large colonies, “++” indicating intermediate growth and/or intermediate sized colonies, and “+” indicating poor growth and/or small colonies. Photographs of representative plates from wild-type *GCD7*, *GCD7* G354V and *GCD7* E239G mutants are presented, with consistent growth phenotypes reported.

**Table 2:** Description of yeast strains used in this study.

Name	Strain	Genotype	Reference
Wild-type	BY4741	*MATa his3Δ1 leu2Δ0 met15Δ0 ura3Δ0*	n/a
SUI2-GFP	BY4741	*MATa his3Δ1 leu2Δ0 met15Δ0 ura3Δ0*	^25^
GCN3-GFP	BY4741	*MATa his3Δ1 leu2Δ0 met15Δ0 ura3Δ0*	^25^
GCD7-GFP	BY4741	*MATa his3Δ1 leu2Δ0 met15Δ0 ura3Δ0*	^25^
GCD1-GFP	BY4741	*MATa his3Δ1 leu2Δ0 met15Δ0 ura3Δ0*	^25^
GCD2-GFP	BY4741	*MATa his3Δ1 leu2Δ0 met15Δ0 ura3Δ0*	^25^
GCD6-GFP	BY4741	*MATa his3Δ1 leu2Δ0 met15Δ0 ura3Δ0*	^25^
GCD6	KAY16	*MAT*α *leu2*-*3 leu2*-*112 ino1 gcd6Δ gcn2Δ::hisG ura3*-*52::P*_*HIS4*_-*lacZ* [*GCD6 LEU2*]	^28, 46^
GCD6 V57G	KAY16	*MAT*α *leu2*-*3 leu2*-*112 ino1 gcd6Δ gcn2Δ::hisG ura3*-*52::P*_*HIS4*_-*lacZ* [*gcd6*-*V57G LEU2*]	^28^
GCD6 I90F	KAY16	*MAT*α *leu2*-*3 leu2*-*112 ino1 gcd6Δ gcn2Δ::hisG ura3*-*52::P*_*HIS4*_-*lacZ* [*gcd6*-*I90F LEU2*]	^28^
GCD6 R284H	KAY16	*MAT*α *leu2*-*3 leu2*-*112 ino1 gcd6Δ gcn2Δ::hisG ura3*-*52::P*_*HIS4*_-*lacZ* [*gcd6*-*R284H LEU2*]	^28^
GCD6 R323P	KAY16	*MAT*α *leu2*-*3 leu2*-*112 ino1 gcd6Δ gcn2Δ::hisG ura3*-*52::P*_*HIS4*_-*lacZ* [*gcd6*-*R323P LEU2*]	^28^
GCD6 G369V	KAY16	*MAT*α *leu2*-*3 leu2*-*112 ino1 gcd6Δ gcn2Δ::hisG ura3*-*52::P*_*HIS4*_-*lacZ* [*gcd6*-*G369V LEU2*]	^30^
GCD6 I413A	KAY16	*MAT*α *leu2*-*3 leu2*-*112 ino1 gcd6Δ gcn2Δ::hisG ura3*-*52::P*_*HIS4*_-*lacZ* [*gcd6*-*I413A LEU2*]	^28^
GCD6 W618R	KAY16	*MAT*α *leu2*-*3 leu2*-*112 ino1 gcd6Δ gcn2Δ::hisG ura3*-*52::P*_*HIS4*_-*lacZ* [*gcd6*-*W618R LEU2*]	^28^
GCD7	H2218	*MATa ura3*-*52 leu2*-*3 leu2*-*112 trp1Δ63::GCN4*-*lacZ TRP1 gcn2Δ gcd7Δ* [*GCD7 LEU2*]	^28, 47^
GCD7 V341D	H2218	*MATa ura3*-*52 leu2*-*3 leu2*-*112 trp1Δ63::GCN4*-*lacZ TRP1 gcn2Δ gcd7Δ* [*gcd7*-*V341D LEU2*]	^28^
GCD7 G354V	H2218	*MATa ura3*-*52 leu2*-*3 leu2*-*112 trp1Δ63::GCN4*-*lacZ TRP1 gcn2Δ gcd7Δ* [*gcd7*-*G354V LEU2*]	^28^
GCD7 E239G	H2218	MATa ura3-52 leu2-3 leu2-112 trp1Δ63::GCN4-lacZ TRP1 gcn2Δ gcd7Δ [gcd7-E239G LEU2]	^28^
GCD7 K300R	H2218	MATa ura3-52 leu2-3 leu2-112 trp1Δ63::GCN4-lacZ TRP1 gcn2Δ gcd7Δ [gcd7-K300R LEU2]	^28^

### Analysis of eIF2B bodies in yeast

Strains with GFP tagged *SUI2*, *GCN3*, *GCD7*, *GCD1*, *GCD2* and *GCD6* were obtained from the GFP fusion library^25^ to monitor eIF2B body formation in wild-type cells (Table 2). Alternatively, a plasmid encoding a GCD7-mRuby2 fusion protein was transformed into the BY4741 strain (Fig. S1 and Table 2). The number of cells with eIF2B bodies (foci or fibers) and the total number of cells were counted in ~250–500 cells per biological replicate. The average percent cells with eIF2B bodies from 3–5 independent experiments is reported, with Student’s t-test used to assess significance.

### Analysis of stress granules and eIF2B bodies in yeast

GFP fusion strains were transformed with a CEN plasmid encoding PUB1-mCherry^24^ using the lithium acetate method^41^. Cultures were grown in synthetic medium lacking uracil, treated and imaged as above. The average percent cells with eIF2B bodies and/or SGs (or neither) from 3–6 independent biological replicates, with ~200–400 cells counted per replicate are shown. A Chi-squared test for independence was performed to assess whether SGs and eIF2B bodies were present in the same cells after 30 minutes of glucose deprivation. Student’s t-test was used to compare the number of cells with SG alone to the number of cells with eIF2B bodies alone.

To determine the relative disassembly rates of SGs and eIF2B bodies, *SUI2*-GFP, *GCN3*-GFP, *GCD1*-GFP and *GCD2*-GFP strains expressing PUB1-mCherry were starved as described above. Glucose repletion was done by addition of 10% glucose in complete minimal medium to the agarose pad overlaying the starved cultures on the microscope stage. Images were acquired every 15 seconds for 15 minutes. Individual cells with eIF2B bodies and/or sSGs were monitored using ImageJ and the time that eIF2B bodies or SGs dissolved was determined for 20–40 individual cells from 2–4 independent experiments. The average time of disassembly and standard deviation is reported, with Student’s t-test used to assess significance.

### Analysis of cytoplasmic pH and eIF2B bodies or stress granules in yeast

A codon-optimized pHuji (a kind gift from Dr. Joel Kralj’s laboratory)^26^ was used to monitor intracellular pH in the GCD1-GFP strain at log phase in selective minimal medium and imaged as described above. Images were acquired by successively monitoring red and green fluorescence to assess pHuji intensity and GCD1-GFP localization (respectively) in glucose starved or replete cells under the same light exposure conditions. The relative intensity of pHuji was assessed using ImageJ. The percent GCD1-GFP tagged cells with eIF2B bodies after glucose repletion on the microscope stage was quantified in each frame from time-lapse images (one frame every 15 seconds for 15 minutes). Student’s t-test was used to assess differences in pHuji intensity from three independent experiments with ~200 cells counted per replicate. Yeast harboring PUB1-mCherry were co-transformed with pHluorin^27^ (a kind gift from Dr. Joel Kralj’s laboratory) and imaged as above to monitor SG disassembly.

### Analysis of macromolecular assemblies in VWMD mutants in yeast

To assess eIF2B bodies, P-bodies and SGs in VWMD mutants, a panel of *GCD6* and *GCD7* yeast harboring mutations analogous to those observed in VWMD patients (generously contributed by Dr. Graham Pavitt^28^) were used (Table 2). These strains were generated by plasmid shuffling to replace the only copy of *GCD6* or *GCD7* with mutant or wild-type *GCD6* or *GCD7* low-copy CEN plasmids under the control of their own promoters^28^, and lacked the *GCN2* gene. A CEN plasmid encoding a GCN3-mRuby2 protein under the GCN3 promoter was used for eIF2B body detection. The number of eIF2B bodies in log phase and under acute glucose deprivation conditions in 200–500 cells per replicate was determined. The average percent cells with eIF2B bodies and the standard deviation is reported from 2–5 biological replicates. Stress granules and P-bodies were assessed in these strains using a plasmid encoding PAB1-GFP and EDC3-mCherry^24^. Yeast were prepared and imaged as above in log phase and glucose deprivation conditions. The average number of cells with SGs in each *GCD7* or *GCD6* mutant strain was determined from 3 biological replicates with 100–900 cells counted per replicate. The average number of P-bodies per cell and standard deviation from 3 biological replicates is reported. The number of P-bodies per cell was determined in 100–150 cells for each biological replicate. Student’s t-test was used to determine significance.

### Plasmid construction

GCN3-mRuby2 and GCN7-mRuby2 CEN plasmids were created using overlap PCR and the NEBuilder HiFi DNA Assembly master mix. Three DNA fragments with overlapping regions were created by PCR using Phusion® High Fidelity DNA polymerase (New England Biolabs) to amplify GCN3 or GCD7 and mRuby2 with overlapping primers. Yeast (BY4741) genomic DNA was extracted for a PCR template as described previously^44^ and a gblock® (Integrated DNA Technologies) of codon-optimized mRuby2 sequence was used. See Table 3 for all primer sequences. A 1626 nt PCR product was generated containing the GCN3 coding sequence and 500 nt upstream of the coding sequence to capture the endogenous promoter. The forward primer “GCN3 PCR 1 Fw” or “GCD7 PCR 1 Fw” was used to create this PCR product overlapped with the vector pRS416, and the reverse primer sequence overlapped with mRuby2. The reverse primer “GCN3 PCR 1 Rv” or “GCD7 PCR 1 Rv” was complementary to 3′ end of the GCN3 or GCD7 coding region lacking a stop codon, to insert mRuby2 into the 3′ region of the GCN3 or GCD7 open reading frame. The second 708 nt PCR product was generated by amplifying the mRuby2 sequence including a stop codon using the “mRuby2 Fw” and “mRuby2 Rv” primers. The third 583 nt PCR product was derived using a forward primer “GCN3 PCR 3 Fw” to the 5′ end of the 3′ untranslated region with homology to the 3′ end of the mRuby2 sequence, and a reverse primer “GCN3 PCR 3 Rv” or “GCD7 PCR 3” 500 nt downstream of the stop codon of the GCN3 or GCD7 open reading frame that overlapped the pRS416 vector.

**Table 3:** Sequences of primers used to create GCN3-mRuby2 and GCD7-mRuby2 in pRS416.

GCN3 PCR 1 Fw:	TTGGGTACCGGGCCCCCCCTCGAGGTCGACGGTATCGATAGACCTAAAGAAAAATAAAAT
GCN3 PCR 1 Rv:	CATGTTTTCTTTAATAAGTTCTTCACCCTTTGAAACATCATACCACATCTTGATTAACTC
mRuby2 Fw:	GTTTCAAAGGGTGAAGAACTTATTAA
mRuby2 Rv:	TTTATATAATTCGTCCATCCCACCCC
GCN3 PCR 3 Fw:	GTAACTTGGGGGGTGGGATGGACGAATTATATAAATAAAAAAAATCACATAATATATGCA
GCN3 PCR 3 Rv:	AGCTCCACCGCGGTGGCGGCCGCTCTAGAACTAGTGGATCGGCCAATATGCAGCATTTGT
GCD7 PCR 1 Fw:	ATTGGGTACCGGGCCCCCCCTCGAGGTCGACGGTATCGATAAGCTTAGGTGCATAACCGA
GCD7 PCR 1 Rv:	TTCTCATGTTTTCTTTAATAAGTTCTTCACCCTTTGAAACCGCCTTATTTTTATCCAAAT
GCD7 PCR 3 Fw:	GTACAGTAACTTGGGGGGTGGGATGGACGAATTATATAAATGATGATGTGTCTTTTGTAC
GCD7 PCR 3 Rv:	GCTCCACCGCGGTGGCGGCCGCTCTAGAACTAGTGGATCCTTTTCTACTTCAGTTCAAGC

### Human Cell Cultures

HeLa cells were maintained in DMEM with 10% FBS and 1% streptomycin/penicillin. HeLa cells were incubated for 24 hours under normoxic or hypoxic (<0.1% O_2_, 20% CO_2_) and acidic (pH ~6) conditions using the Anaeropack system (Thermo Fisher Scientific) for immunofluorescence experiments. Immortalized lymphoblasts derived from two patients with VWMD (GM20073 and GM20074) and two age, sex and ethnicity-matched controls (GM16118 and GM05377) described in^18^ were obtained from Coriell Biorepository. Lymphoblasts were grown in RPMI 1640 supplemented with 15% FBS and 1% streptomycin/penicillin.

### Indirect immunofluorescence microscopy

P-bodies were detected in lymphocytes adhered to chamber slides using poly-L-lysine. EIF2B4 localization was assessed in HeLa cells. Cells were fixed with 4% paraformaldehyde and permeabilized with triton-X-100. Indirect immunofluorescence was performed using antibodies to DDX6 (DEAD-box helicase 6; MBL PD009) for P-bodies or eIF2B4 (Santa Cruz Biotechnology, sc-28855) and donkey anti-rabbit secondary antibodies conjugated to AlexaFluor 594 (Jackson Immunoresearch Laboratories, Inc) and ProLong Gold Antifade mounting medium (Thermo Fisher Scientific). Microscopy was done using a wide field DeltaVision Elite microscope with PCO Edge sCMOS camera using a 100x objective, and images deconvolved. The number of P-bodies per cell was counted using Fiji^45^. Results represent the average of four independent experiments and significance was assessed by Student’s t-test.

## Electronic supplementary material

Video S1

Video S2

Supplementary Information

## Data Availability

No datasets were generated or analyzed during the current study.
