# Supplementary material for: Analysis of eIF2B bodies and their relationships with stress granules and P-bodies
Source: Sci Rep. 2018 Aug 16;8:12264. doi: 10.1038/s41598-018-30805-y (PMC6095920; doi:10.1038/s41598-018-30805-y)
Supplement: Supplementary file 3 — Supplementary Information [file 41598_2018_30805_MOESM3_ESM.pdf]

Supplementary Materials

Analysis of eIF2B bodies and their relationships with stress granules and P-bodies

Stephanie L. Moon and Roy Parker

Figure S1-S5

Titles and captions of all videos

Video S1. Disassembly of eIF2B bodies occurs rapidly following glucose repletion after starvation. Yeast with endogenous GFP-tagged GCD2 were grown to log phase and starved of glucose for 30 minutes to induce eIF2B body formation. A 1% agarose pad made with PBS was overlaid on a drop of the culture on a cover glass. A solution of 10% glucose in growth medium was added to the agarose pad and GFP was imaged every 15 seconds for 15 minutes on a spinning disc confocal microscope (100x).

Video S2. EIF2B body disassembly occurs as cytoplasmic pH increases upon glucose repletion following acute glucose starvation. Yeast encoding GFP-tagged GCD1 were transformed with a plasmid encoding the red fluorescent cytoplasmic pH sensor pHuji. A log phase culture was starved of glucose for 30 minutes and then imaged under an agarose pad using a spinning disc confocal microscope at 100x. Images were acquired every 15 seconds for 15 minutes after a solution of 10% glucose was added to the agarose pad for glucose repletion.

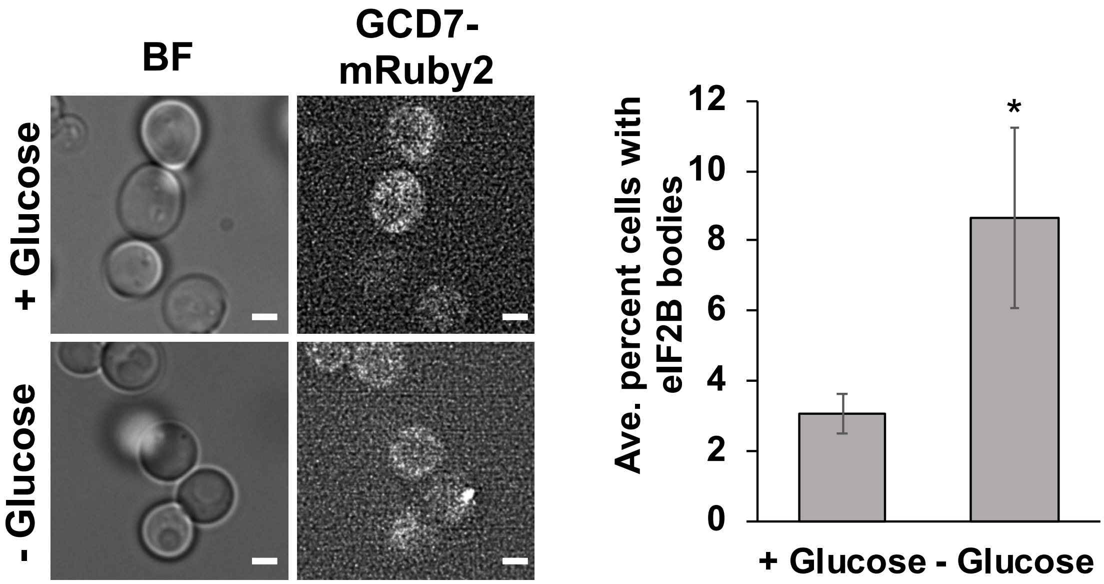

Figure S1. Imaging yeast under coverslips confirms EIF2B bodies are induced upon glucose deprivation stress. Yeast transformed with a plasmid encoding GCD7-mRuby2 were grown to log phase then incubated in either complete synthetic medium ("+ Glucose") or complete synthetic medium lacking glucose ("- Glucose") for 30 minutes. Cells were imaged under glass coverslips on a DeltaVision Elite widefield microscope in the brightfield ("BF") or red ("GCD7-mRuby2") channels. At left are representative images with scale bars indicating 2 μm. At right the percent cells with eIF2B bodies from 3-5 frames (521 cells in "+ Glucose" and 92 cells in "– Glucose" total) is shown.

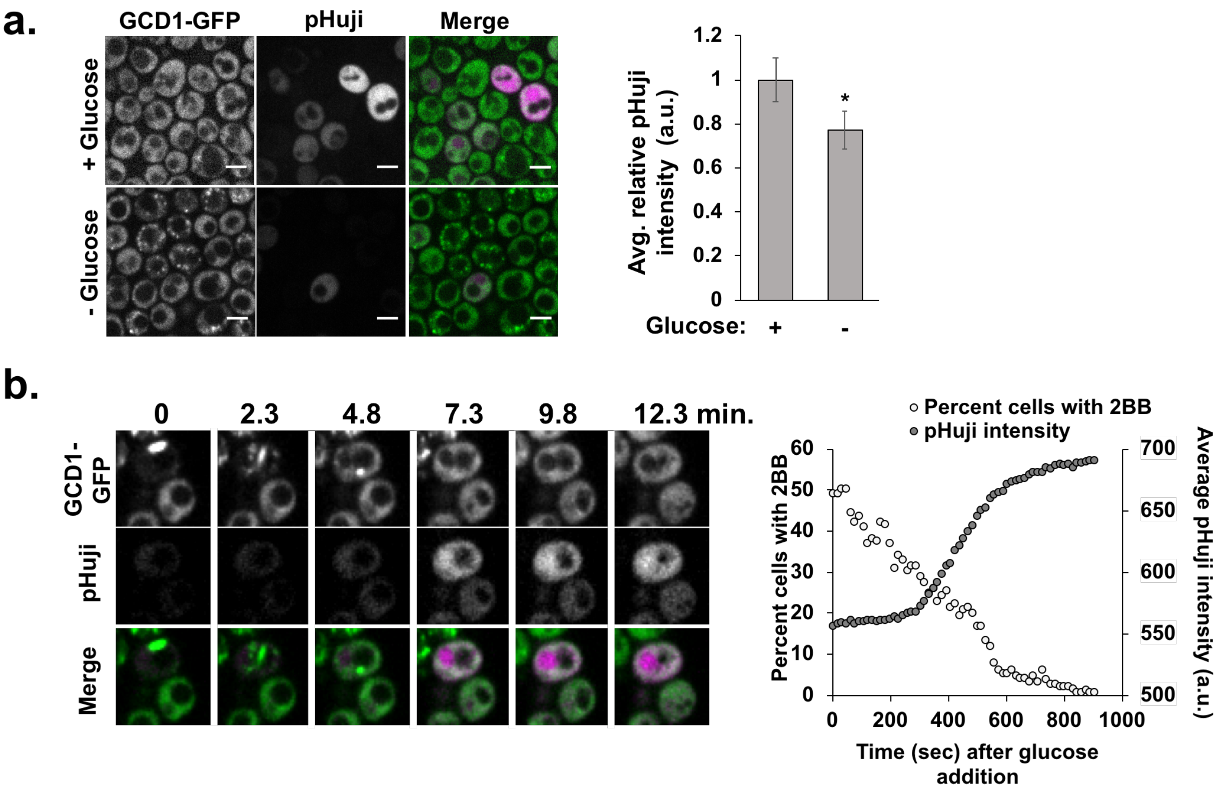

Figure S2. eIF2B bodies form in association with cytoplasmic acidification during glucose deprivation stress and dissolve as pH increases with glucose repletion. Yeast with GFP tagged GCD1 were transformed with a plasmid encoding a red pH sensor "pHuji". A) Yeast grown to log phase were starved of glucose or kept in glucose replete medium for 30 minutes then imaged under a 1% agarose pad on a spinning disc confocal microscope (100x). Representative images are shown and the graph represents the average relative pHuji signal intensity +/- S.D. from ~200 cells per replicate from three independent experiments. Scale bars are 5 μm. B) Yeast starved of glucose for 30 minutes were placed under 1% agarose pad and a solution of 10% glucose was added for glucose repletion. A montage (showing every 10 frames for 12.3 minutes) depicting disassembly of 2B bodies and increased pHuji intensity indicative of pH increase is shown at left. At right, quantification of bulk pHuji intensity (dark gray dots) and the percent cells with 2B bodies (light gray dots, "2BB") in ~200 cells following glucose repletion at time 0 with pHuji and GFP imaged every 15 seconds for 15 minutes.

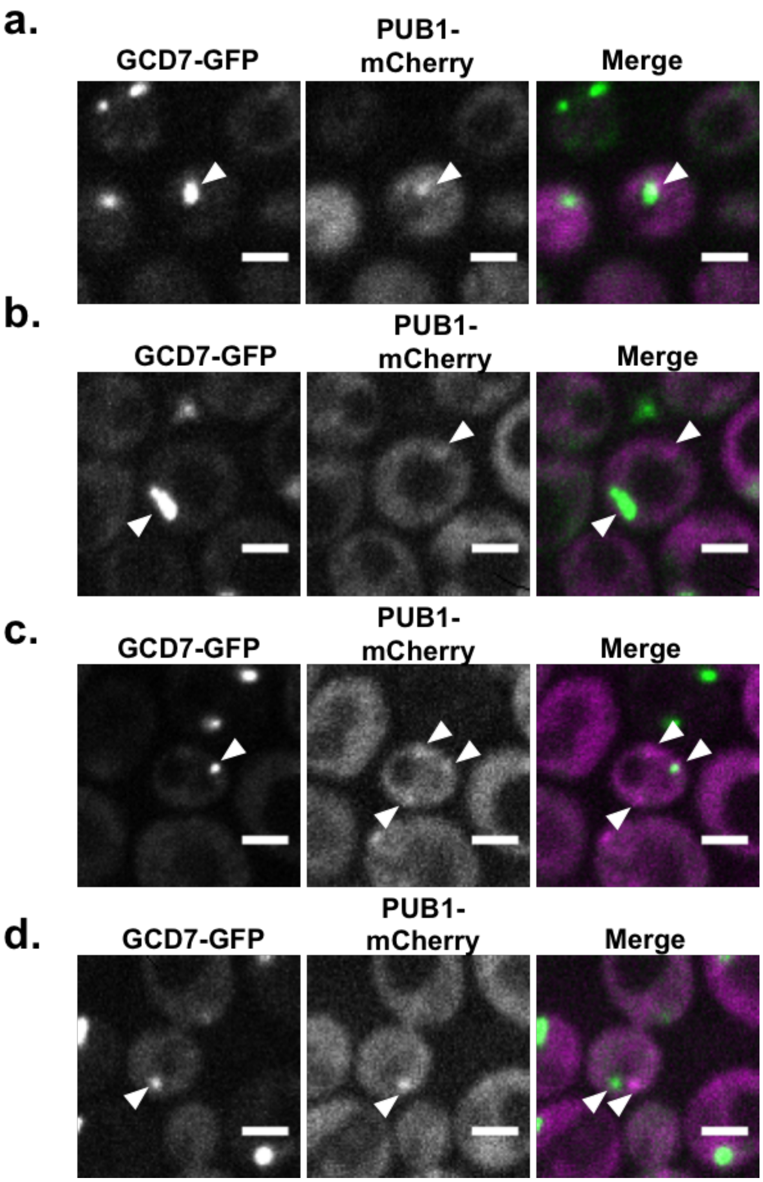

Figure S3. Stress granules and eIF2B bodies can either colocalize (a), be present at opposite ends of the cell (b), or both (c), and can be observed adjacent to one another (d). The GCD7-GFP yeast strain expressing the stress granule marker PUB1-mCherry was grown to log phase and starved of glucose for 30 minutes, then imaged as in Figure 2. Arrows indicate the location of eIF2B bodies (GCD7-GFP foci, green) or stress granules (PUB1-mCherry foci, magenta). Scale bars indicate 5 μm.

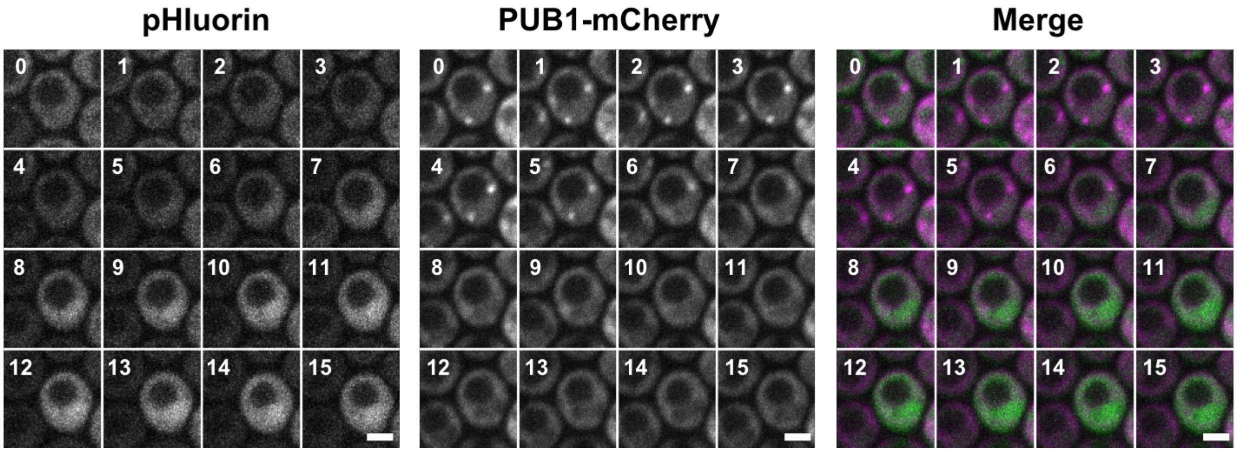

Figure S4. Stress granules induced by glucose repletion dissolve immediately upon glucose repletion. Yeast transformed with plasmids encoding the green fluorescent cytoplasmic pH sensor super ecliptic pHluorin (green) and the red stress granule marker PUB1-mCherry (magenta) were grown to log phase and starved of glucose for 30 minutes. Cells were then imaged under agarose pads. At time ‘0’, complete minimal medium with 10% glucose was added to the agarose pad for repletion, and imaging done every 15 seconds for 15 minutes. Montages depict one frame every minute for 15 minutes (upper left corner).

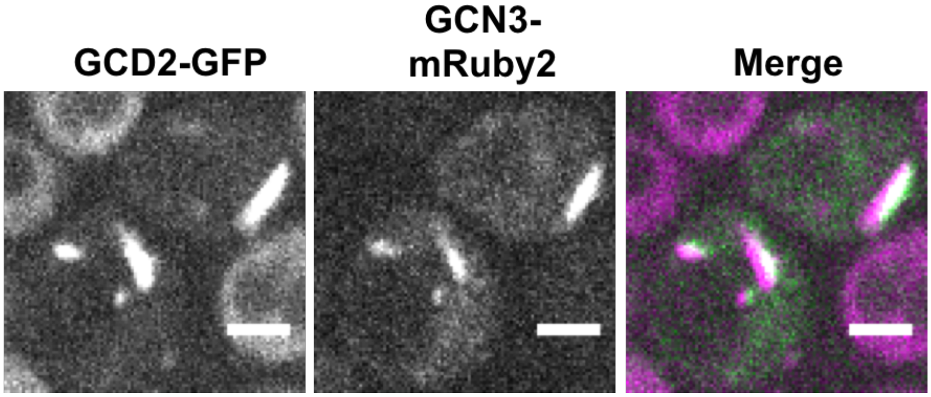

Figure S5. An mRuby2-tagged GCN3 protein encoded on a plasmid co-localizes with endogenously tagged GCD2-GFP. The GCD2-GFP yeast strain was transformed with a plasmid encoding GCN3-mRuby2, grown to log phase and imaged after 30 minutes of glucose deprivation. GCD2-GFP (green) and GCN3-mRuby2 (magenta) were imaged on a spinning disc confocal microscope at 100x under 1% agarose pads.
